# Enhanced nursing self-awareness and pharmacotherapy knowledge-base: peer-teaching and nursing/pharmacy interprofessional education

**DOI:** 10.1080/10872981.2020.1814551

**Published:** 2020-08-30

**Authors:** Brecon Powell, Kim D. Jardine, Michelle Steed, Jennifer Adams, Barb Mason

**Affiliations:** a Idaho State University College of Pharmacy, Pocatello, ID, USA; b Idaho State College of Nursing, Pocatello, ID, USA; c Idaho State University College of Pharmacy, Meridian, ID, USA

**Keywords:** Interprofessional, peer-teaching, knowledge-base, nursing, pharmacotherapy, pharmacy

## Abstract

Peer-teaching and interprofessional education can help students define individual healthcare roles, establish improved interprofessional relationships, and improve overall knowledge-base. Peer-teaching was provided by student pharmacists completing Advanced Pharmacy Practice Experiences (APPEs) for nine weeks to undergraduate student nurses. 4 student pharmacists and 25 student nurses participated in this study. Knowledge-base was measured with quizzes that were pre and post educational intervention. Confidence, self-awareness, and interprofessional perception were assessed using a modified ISVS-9A. Data were analyzed using a paired t-test. The mean difference between pre and post-knowledge-based tests averaged an improvement of 3.15 points (out of an average of 10.8 points per test) showing an overall improvement (p < 0.003). A total of 25 nursing students completed a pre and post-ISVS-9A questionnaire. The mean difference between the pre and post-questionnaire items showed an average improvement of 0.87 points (p < 0.05). Interprofessional peer-teaching showed overall improvement in student nurse knowledge-base and perceived value of and socialization to interprofessional care. This study required no financial funding, and there are no conflicts of interest to disclose.

## Introduction

In order to meet the public’s increasingly complex health care needs, the educational experience is shifting from one in which health profession students are educated in silos to one that fosters collaboration, communication, and a team approach to providing care [1]. Interprofessional collaboration allows different health care professionals to bring their unique skills together as a team to provided efficient and effective patient care [2]. With an increasingly complex patient population receiving healthcare services, a holistic approach is required to ensure patient safety, guideline-driven therapy, and improved patient outcomes [3]. Involving students of different medical disciplines in interprofessional peer-teaching experiences helps develop cohesive relationships for the future [4]. Collaborative learning by students at the practice site during experiential learning is a promising practice that supports the creation of an institutional environment in which interprofessional collaborations can thrive and create a culture of interprofessional collaboration. (Robert Wood Johnson Foundation-Lessons from the Field: Promising Interprofessional Collaborative Practices). One of the most recent significant efforts by the accrediting body twenty-five-member group: The Health Professions Accreditors Collaborative (HPAC) has been the development of a guidance document to assure that students in foundational and graduate education programs are prepared for interprofessional collaborative practice upon graduation [5. This document encourages an increase in communication and collaboration on expectations related to quality interprofessional education and is supportive of the nursing pharmacy interprofessional education experience described in this paper. Nurses play a vital role in patient medication management that can affect patient outcomes (6]. Medication-related responsibilities for nurses include: managing physician medication orders; administration of medications; medication reconciliation in transitions of care; monitoring for allergies and side effects; adjusting drip rates and drug titrations; and medication education. In order to fulfill these responsibilities effectively and efficiently, nurses should have a basic knowledge of pharmacotherapy, drug safety, and drug administration [7]. Nurses should have a sound understanding of their occupation identity and that nurses and pharmacists can work synergistically together to improve overall patient care [8]. For this reason, this study measured the changes in pharmacotherapy knowledge-base, self-confidence, and value of and socialization to interprofessional care of student nurses receiving pharmacotherapy peer-education from student pharmacists completing general medicine Advanced Pharmacy Practice Experiences (APPEs). This study went through the XXX IRB process and was found to be exempt on the basis of being educational research.

## Methods

### Participants

Undergraduate student nurses and final-year student pharmacists completing general medicine Advanced Pharmacy Practice Experiences (APPEs) participated in this study. Two different sets of student pharmacist pairs (2 females, 2 males) participated in the teaching of these student nurses. A total of 25 student nurses (19 females, 6 males) participated in this study and were initially assigned to complete experiential education hours at the hospital by their educational institution. Student nurse attendance varied week to week based on their preceptors’ permission to attend IPE discussions and individual experiential education schedules. All participants have been anonymized. One faculty member from nursing and pharmacy were present during each weekly discussion. Data was collected from the students that completed both pre and post-knowledge-based tests, and any student that did not complete both tests for a specific topic was excluded from data collection. The weekly participation numbers can be found in Table 1. All 25 student nurses completed the pre and post ISVS-9A surveys.

**Table 1.** Pharmacotherapy pre and post-quiz data.

Topic	% of n	Pre-test Average	Post-test Average	Mean Difference	Standard Error of Mean Difference	T-statistic	P-value
Pulmonary therapeutics	9	6.8/10	9.3/10	2.4	0.376	6.47	<0.00001
Infectious disease	18	4.2/10	7.8/10	3.6	0.672	5.35	<0.00001
Cardiovascular medications #1	19	4.6/12	8.6/12	3.9	0.346	11.37	<0.00001
Cardiovascular medications #2	16	5.4/12	9.1/12	3.75	0.513	7.31	<0.00001
Diabetes therapeutics	16	5.4/13	9.75/13	4.1	0.506	8.12	<0.00001
Pain management	17	7.4/12	10.3/12	2.9	0.432	6.81	<0.00001
Antidepressants & antipsychotics	20	5/10	8.1/10	3.1	0.324	9.51	<0.00001
GERD and IBD	22	4.6/11	7.6/11	3.6	0.275	13.27	<0.00001
Geriatrics & Beer’s List	19	4.4/8	5.42/8	1	0.367	2.74	0.003

### Materials and procedure

Final year student pharmacists completing their general medicine APPE provided nine weekly pharmacotherapy reviews for undergraduate student nurses. The pharmacotherapy reviews included overviews of common disease states, medication treatment options, and case discussions. The student nurses completed a pre and post-knowledge-based quiz at the beginning and end of each topic discussed. Patient cases that were in line with the weekly topic were discussed between pharmacy and nursing students. A modified Interprofessional Socialization and Valuing Scale (ISVS-9A) was administered to the student nurses at the beginning and end of the nine weeks [9].

### Objectives

The primary outcome of this study was to compare student nurses’ knowledge-base, self-confidence, and socialization to and value of interprofessional interaction at baseline and after nine weekly pharmacotherapy reviews from student pharmacists.

### Activity description

Data was collected and analyzed over the course of four months. APPE student pharmacists that were completing their general medicine APPEs were responsible for preparing nine weekly educational reviews for undergraduate student nurses. A teaching outline was used and approved by faculty to cover the basics of pathophysiology, the most commonly used medications to treat a specific disease, brand and generic names, mechanisms of action, side effects, drug administration, and common drug interactions. These topics can be modified to align with the current didactic lectures the student nurses are learning in their curriculum. Educational topics included: pulmonary medications, antibiotics, cardiovascular medications, diabetes therapeutics, pain management, antidepressants and antipsychotics, gastroesophageal reflux disease and inflammatory bowel disease, and geriatric considerations. Each educational discussion lasted for 60–90 minutes, allowing the group to also discuss pertinent patient cases.

A formative knowledge-based pre-test was provided at the beginning of each class, and the summative post-test specific to each educational topic was completed at the end of each class. The tests were composed of 8 − 12 questions that covered the highlights of each topic discussion. Each student nurse’s post-test was compared against their pre-test score to measure overall improvement. The pharmacotherapy-based topics and quizzes were selected based on previous feedback from student nurses. Test questions were developed by the APPE student pharmacists following their instructional preparation, and approved by pharmacy and nursing faculty.

The modified ISVS-9A helped assess views and perceptions at a point in time in regards to interprofessional activities with a scale of 0–7 (Table 2). The final item of this survey was added to assess student nurse socialization to and perception of interprofessional relationships in regards to pharmacy and patient care. This survey was provided to the 25 student nurses on the first day of the nine weeks prior to the start of any interprofessional instruction. APPE student pharmacists, pharmacy and nursing faculty followed up with each of the student nurses at the end of the 9 weeks to collect the post modified ISVS-9a survey.

**Table 2.** Modified ISVS-9A.

Modified ISVS-9A criteria	n	Average Pre-score (out of 7 points)	Average Post-score (out of 7 points)	Mean difference between pre and post surveys	Standard Error of Mean	T-statistic	P-value
1. I am able to share and exchange ideas in a team discussion	25	4.92	6	1.08	0.237	4.55	<0.0005
2. I have gained an enhanced perception of myself as someone who engages in interprofessional practice	25	4.88	6.2	1.32	0.214	6.17	<0.0005
3. I feel comfortable in speaking out within the team when others are not keeping the best interests of the client in mind	25	5.16	6.12	0.96	0.241	3.98	<0.0005
4. I believe the best decisions are made when members openly share their views and ideas	25	6.36	6.72	0.35	0.207	1.74	<0.05
5. I feel comfortable in describing my professional role to another team member	25	5.36	6.32	0.96	0.187	5.14	<0.0005
6. I have gained an enhanced awareness of roles of other professionals on a team	25	5.44	6.6	1.16	0.16	7.25	<0.0005
7. I have gained an appreciation for the importance of having the client and family as members of a team	25	6	6.76	0.76	0.210	3.61	<0.005
8. I am comfortable engaging in shared decision making	25	5.52	6.2	0.68	0.263	2.59	<0.01
9. I feel comfortable in accepting responsibility delegated to me within a team	25	5.6	6.52	0.92	0.244	3.76	<0.0005
10. I feel nursing and pharmacy have a synergistic relationship that can improve patient care*	25	6.44	6.96	0.52	0.131	3.98	<0.0005

*Item added to original ISVS-9a

### Data analysis

Continuous data was collected from pre and post quiz scores and ISVS-9A rankings were compared using a paired t-test analysis. The collected data was normally distributed, and a p < 0.05 was considered to be statistically significant. The study population was a random sample from the overall nursing class.

## Results

Each pharmacotherapy topic showed knowledge-based improvements in the average scores of the post-tests vs pre-tests (paired *t* test, p < 0.003). There was one student per test that showed no improvement between pre and post-tests. Infectious disease and geriatrics were the two topics that had students (two students per topic) that did worse on a post-test vs a pre-test. Table 1 shows the pre- and post-test averages, mean differences, and p-values from the data collected.

Each item from the Modified ISVS-9A showed improvement on the post-survey vs. the pre-survey. An average was taken from each criterion in the pre-survey and compared against the average of the post-survey (paired *t* test, p < 0.05). Table 2 shows the statistically significant differences between the pre- and post-ISVS9A submission averages.

## Discussion

With the increasing complexity of healthcare today, nurses and pharmacists have a shared interest in achieving the best care for their patients. To do so, these two professions must work together to ensure appropriate medication therapies are provided to those they are caring for. An interprofessional approach to patient care can improve outcomes and patient perception of care [2]. Teaching pre-licensure students while engaged in a collaborative learning environment could assist in readiness for entry-level clinicians to function on an interprofessional team. (Anderson, 2011) and meet expectations of health care employers [10].

[11]. Strong interprofessional teams that recognize individual roles and strengths, can help reduce drug errors and potentially improve overall job satisfaction [3,12]. A variety of learning methods have been utilized for interprofessional education. Ideal methods are active, interactive and develop and reinforce skills for collaborative practice. Through face to face practice site learning, students’ needs are met and classroom learning reinforced in the continuum of interprofessional learning [13]. For these reasons, this study looks at an educational peer-teaching approach to improve interprofessional relationships and knowledge base to potentially improve patient care in the future.

Pharmacotherapy can be a challenging topic for many healthcare professionals as they enter the workforce, including nurses. This study shows a benefit for students to have interprofessional discussions regarding best practice therapeutics. These types of discussions and peer-teaching should continue once students enter interprofessional practice. There will always be overlap in responsibilities among healthcare professionals, but a clear awareness of each health profession’s roles can streamline how we provide patient care. Nurses and pharmacists developing strong relationships allow for safe interprofessional discussion to verify appropriate medication management is always used. A lack of communication and poor knowledge-base can increase medication errors which is one of the top errors in health care leading to patient harm [13]. While the majority of nursing students showed an improvement in their knowledge-base, this study demonstrated a small number of students that did not improve their pharmacotherapy knowledge-base, demonstrating the importance of developing strong relationships with pharmacists with pharmacotherapy expertise. In addition, this study, pre-ISVS9A survey showed that this this sample of students nurses had a strong base-belief in the synergistic relationship between pharmacist and nurses in improving patient care. Over the course of this study, these beliefs and perceptions improved even further [15].***

Limitations of the study include the small sample size, and the quizzes were low-stakes and were not included in the student’s overall grade; therefore, the commitment to high performance may have been lacking. Even though the number of students in attendance varied, there was still a significant improvement in pharmacotherapy quiz scores each week. There are limitations with using a pre and post-test and survey. With the pre and post-test approach, improvements in knowledge base shown in the post-test can be attributed to short-term memory retention, and it does not show an accurate representation of long-term memory retention. The post-test was the same as the pre-test, which brought the student nurse’s attention to what items to remember during the discussion. With a pre and post-survey, it could be said that familiarity breeds congeniality which could be responsible for the positive changes in the post-survey responses. The overall goal is that familiarity between healthcare workers breeds improved patient care through the development of strong interprofessional relationships of trust, communication, and an understanding of each other’s roles.

## Conclusions

Weekly interprofessional peer-taught discussions provided by final-year student pharmacists helped increase nursing pharmacotherapy knowledge-base, awareness of self-roles and roles of other healthcare professionals, confidence in engaging in interprofessional practice, ability to communicate ideas in a group to improve patient outcomes and enhance relationships between pharmacy and nursing. Interprofessional peer-teaching can have a positive impact on knowledge-base and interprofessional relationships, and there is a need for these peer-teaching experiences to be continued in practice. Further studies will be required to show the effect of interprofessional peer-teaching in reducing medication errors.
